# Occurrence, Formation and Function of Organic Sheets in the Mineral Tube Structures of Serpulidae (Polychaeta, Annelida)

**DOI:** 10.1371/journal.pone.0075330

**Published:** 2013-10-07

**Authors:** Olev Vinn

**Affiliations:** Department of Geology, University of Tartu, Tartu, Estonia; Australian Museum, Australia

## Abstract

A scanning electron microscopy study of organic sheets in serpulid tube mineral structures was carried out to discern their function, formation and evolution. The organic sheets may have some taxonomic value in distinguishing the two major clades of serpulids previously identified. The organic sheets in the mineral tube structure occur only in certain taxa belonging to clade A, but not all species in clade A have them. Organic sheets are best developed in genus *Spirobranchus*. One could speculate that organic sheets have evolved as an adaption to further strengthen the mechanical properties of the tubes in clade A, which contains serpulids with the most advanced mineral tube microstructures. The organic sheets are presumably secreted with at least some mineral phase.

## Introduction

Serpulids are marine polychaete tubeworms that dwell in all latitudinal and depth zones of the ocean [1]. Serpulids are the only polychaete tubeworms with exclusively calcareous tubes. They have a long evolutionary history from the Middle Triassic to the Recent [2]. Serpulids are important calcifiers, especially in the temperate seas where they construct small reefs [3], [4]. They are vulnerable to the ongoing modern ocean acidification [4], [5].

Serpulids have the most advanced biomineralization system among the annelids [6], [7]. Serpulids can build tubes of aragonite, calcite or a mixture of both of these minerals [6], [4]. Serpulid skeletal microstructures are similar to those found in a variety of invertebrate phyla and are comparable to those of cnidarians and arthropods in diversity [8]. Serpulids form two major clades that have characteristic microstructural complexities. Oriented microstructures, either simple or complex, occur only in one clade (A) of serpulids, while isotropic microstructures are found in both clades (A and B) [9].

Serpulid tubes contain both soluble (in EDTA) and insoluble organic matrices [7]. The major components of the soluble organic matrix are carboxylated and sulfated polysaccharides [7]. Minority components of the soluble organic matrix are various amino acids, such as aspartic acid, glutamic acid, glycine and proline. Tanur et al. [7] found that organic sheet structures observed via SEM in *H*. *dianthus* could possibly be composed of polysaccharides. Sulfated and carboxylated polysaccharides have been found to influence the process of biomineralization. They have the capability to bind cations, which can play a role in the nucleation process [10].

The inner organic tube lining of serpulids has been studied for its role in biomineralization [7], [11]. However, the details of the occurrence, function and formation of the organic sheets inside the mineral structures of serpulid tubes remain poorly known. Their role in serpulid biomineralization is also not well understood. This study seeks to shed light on the taxonomic occurrence, evolution, function and formation of organic sheets in serpulid tubes.

The main questions to be addressed are:

1. Are the organic sheets secreted together with the mineral phase of the tube (differentiating after deposition on growth surface) or separately (during a separate event) from a mucus of different composition?
2. What is the function of inner organic sheets?
3. Do the organic sheets occur in both aragonitic and calcitic structures?
4. Is there any correlation between the structural type and occurrence of organic sheets?
5. Are there taxonomic differences in the abundance, morphology and dimensions of organic sheets?

## Materials and Methods

43 serpulid species (23 belonging to clade A and 20 belonging to clade B) collected worldwide from various depth zones were studied for organic sheets in the mineral structure of their tubes (Table 1, Table S1). Specimens were donated by the Netherlands Centre for Biodiversity, Naturalis for the study of serpulid skeletal microstructures. Voucher specimens of the studied material are deposited at the Netherlands Centre for Biodiversity, Naturalis. The collections were accessed under the SYNTHESYS NL-TAF-111 project titled: “Tube microstructure in serpulid polychaetes”.

**Table 1:** Serpulid species with organic sheets and their tube microstructures.

Species	Clade	Tube layers	Occurrence of organic sheets by tube layers/thickness of sheets	Material studied (localities and depths, when known)
*Crucigera websteri*	A	SPHP/IOP/LF/SPHP	**IOP/LF/SPHP** (0.4–0.7 µm)	V.Pol.3589 Surinam, 60 m
*Crucigera zygophora*	A	SPHP/IOP/SIOP	Possible occurrence	V.Pol.3287 Canoe Bay, Alaska, USA, 8 m
*Floriprotis sabiuraensis*	A	IOP/LF/SLF	**LF/SLF** (0.6–0.7 µm)	V.Pol.3929 Shimoshima Island, Amakusa, Japan, 10 m
*Galeolaria hystrix*	A	LF/SIOP	**LF/SIOP** (0.6–0.7 µm)	V.Pol.3576 New Zealand, Queen Charlotte Sound, 1–2 m
*Hydroides dianthus*	A	SPHP/IOP/LF	LF (0.6–07 µm)	V.Pol.3661 USA, Anna Maria Island, FL, 2 m
*Hydroides spongicola*	A	SIOP	**SIOP** (0.5–0.7 µm)	V.Pol.3584 Netherlands Antilles, Curaçao, 7 m
*Neovermilia sphaeropomatus*	A	LF	Possible occurrence	V.Pol.3274 New Zealand, Cape Saunders, 10 m
*Placostegus tridentatus*	A	SP	Possible occurrence	V.Pol.1105 Norway, Bergensfjord
*Pyrgopolon ctenactis*	A	SOSIOP	Possible occurrence	V.Pol.4969 Netherlands Antilles, Bonaire, 15 m
*Serpula vermicularis*	A	LF	LF (0.5–0.6 µm)	V.Pol.3780 Ireland, Ardbear Lough, 20 m
*Spirobranchus americanus*	A	IOP/LF	IOP/LF (0.6–0.7 µm)	ZMA V.Pol. 5009 Ensign, trawled 10 miles east of Bony, R’4’ (Knuckle Bony) off Cape Lookout Shoals, 10–20 m, U.S.A.
*Spirobranchus giganteus*	A	OF/SIOP	**OF/SIOP** (0.1–0.3 µm)	Netherlands Antilles, Curaçao, 6 m
*Spirobranchus kraussii*	A	SPHP/IOP/LF/SIOP	**IOP/LF/SIOP** (0.3–0.4 µm)	V.Pol.4748 Teluk Slawi, Indonesia, 0.5 m
*Spirobranchus triqueter*	A	LF	LF (0.4–0.5 µm)	Sweden, Tjärnö, 10 m
*Vitreotubus digeronimoi*	A	SP	SP	V.Pol.4308, Seychelles, Platte Island, Sta. 795, 600 m

Isotropic structures: HAC – homogeneous angular crystal structure, FH – fine grained homogeneous structure, IOP – irregularly oriented prismatic structure, IOPL – irregularly oriented plate-like structure, RHC – rounded homogeneous structure, SIOP – spherulitic irregularly oriented prismatic structure. Semi-oriented structures: SOIOP – semi-ordered irregularly oriented prismatic structure, SOSIOP – semi-ordered spherulitic oriented prismatic structure. Oriented prismatic structures: RRP- regularly ridged prismatic structure, SP – simple prismatic structure, SPHP – spherulitic prismatic structure. Oriented complex structures: LF – lamello-fibrillar structure, OF – ordered fibrillar structure, SLF – spherulitic lamello-fibrillar structure. Tube layers are ordered from outside (left) to lumen (right). Occurrence of multiple organic sheets indicated by bold letters.

Serpulid tubes were cut using a small electrical saw and a razor blade. Oriented tube portions were mounted in Canada balsam for machine grinding. All sections of tubes were polished and etched in a 1% solution of acetic acid for 1 minute, then the preparations were gold-sputter coated prior to SEM study. SEM investigations were performed on a Hitachi S-4300 SEM, equipped with an Inca EDX system, at the Swedish Museum of Natural History, Stockholm and on a Zeiss 940D SEM, equipped with SAMx SDD EDX, at the Department of Geology, University of Tartu. The beam was operated at 5 to 10 kV and 1 nA. Five to fifteen millimeter long longitudinal sections, and one to three cross sections of each serpulid species were studied. Some samples were repolished and treated with a 1∶1 mixture of 25% glutaraldehyde and 1% acetic acid, to which Alcian blue was added (Mutvei solution) before additional SEM examination [12]. This treatment helps to fixate organic-rich parts of the tubes. A few samples were additionally repolished and bleached with NaClO to remove organics without affecting the mineral part of the tube before the SEM study.

The thickness of sheets was measured from the mineral layer below to the mineral layer above the sheet as the sheets themselves were partially decomposed due to sample treatment. Thus all the sheet thicknesses given here are approximate. In cases where the sheets were not unequivocally identifiable, possible occurrences were marked. In cases where the absence of sheets was not unequivocally determined, sheets possibly absent were marked. In certain cases the thickness of sheets was measured on SEM photos with a ruler.

## Results

**Figure 1:**
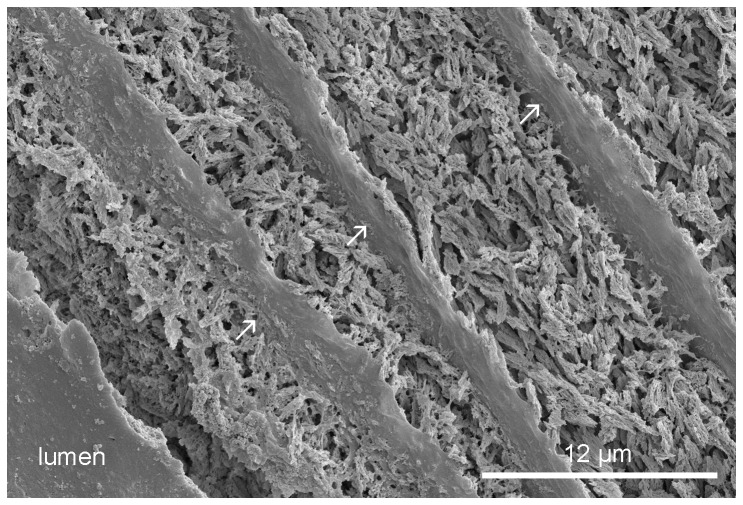
*Spirobranchus kraussii*, longitudinal section of the tube. The inner part of the tube wall, showing multiple organic sheets (arrows).

**Figure 2:**
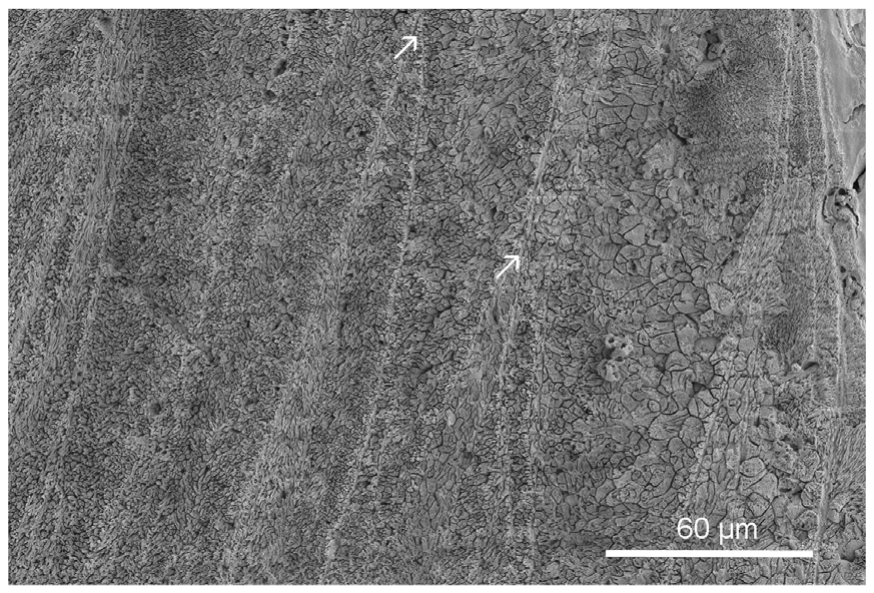
*Floriprotis sabiuraensis*, longitudinal section of the tube. The inner part of the tube, showing merging organic sheets (arrows).

Organic sheets occur or possibly occur in 15 of the 43 serpulid species studied (Table 1). Multiple organic sheets occur in six of 15 species with organic sheets (Figure 1). In the six species with multiple organic sheets there can be up to eight organic sheets per 50 µm of tube wall. In the other species only a few organic sheets occurred in the tube wall (Table 1). Organic sheets occur only in serpulids belonging to clade A, while species of clade B have no organic sheets in their mineral tube structures. All studied species (N = 4) of the genus *Spirobranchus* have well developed organic sheets. In some species (*Crucigera websteri*, *Floriprotis sabiuraensis*) organic sheets are more common in the inner part of the tube wall. The organic sheets can cover lens-like mineral deposits and merge with each other (Figure 2).

**Figure 3:**
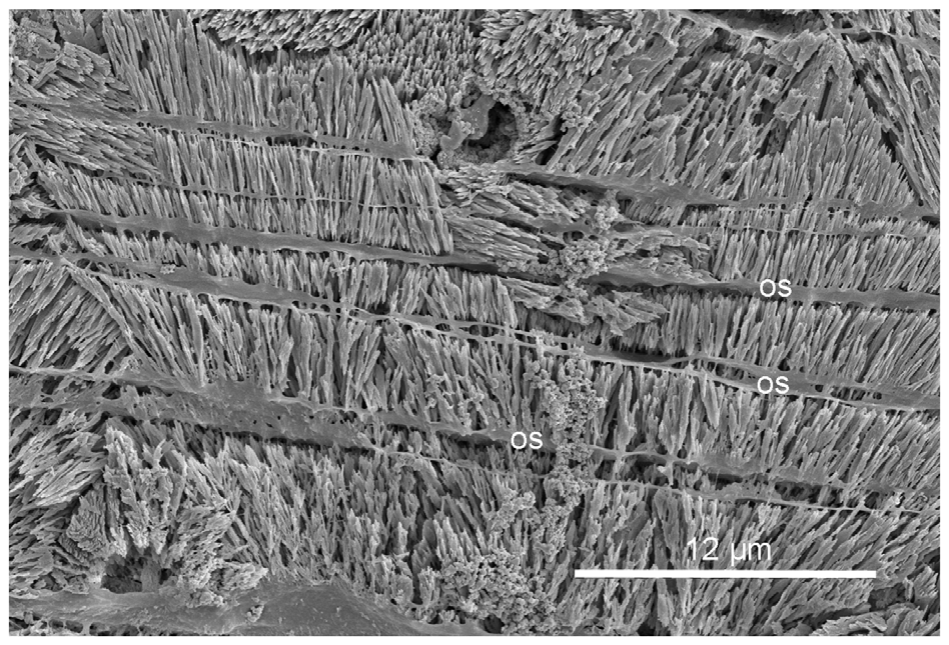
*Spirobranchus giganteus*, longitudinal section of the tube. The inner part of the tube, showing epitaxial growth of spherulitic prisms through multiple organic sheets (os).

**Figure 4:**
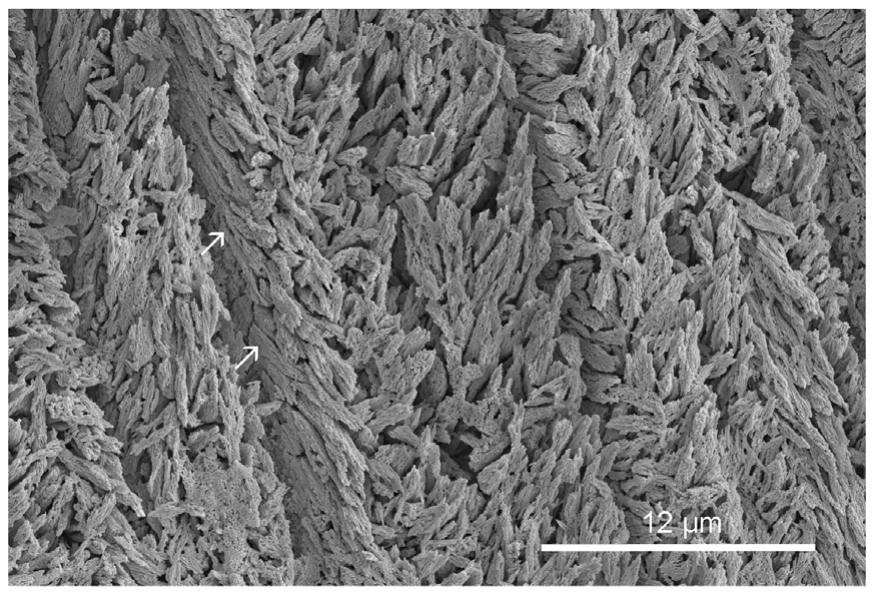
Floriprotis sabiuraensis. Bleached (NaClO) transverse section through SLF structure. Arrows point to the flat upper surface of spheruitic lamello-fibrillar structure in contact with organic sheets.

The occurrence of organic sheets does not depend on the type of tube structure. Organic sheets can occur in isotropic structures such as irregularly oriented prismatic (IOP) and spherulitic irregularly oriented prismatic (SIOP), in oriented prismatic structures such as spherulitic prismatic (SPHP), and in complex oriented structures such as lamello-fibrillar (LF), spherulitic lamello-fibrillar (SLF) and ordered fibrillar (OF) structures. Prismatic crystals were observed to grow epitaxially through the organic sheets in *Crucigera websteri*, *Floriprotis sabiuraensis*, *Spirobranchus giganteus* (Figure 3) and *S*. *kraussii*. *Floriprotis sabiuraensis* has an extremely flat growth surface of crystals in contact with the organic sheets in SLF structure (Figure 4).

The thickness of organic sheets varies from 0.1 to 0.7 µm. The dimensions of the organic sheets do not depend on the type of tube microstructure. There is some taxonomic variability in the organic sheet thicknesses (Table 1). There is no remarkable difference in the morphology of organic sheets between the studied taxa.

The organic sheets could occur both in calcitic (*Palcostegus tridentatus*, *Spirobranchus triqueter*, *Serpula vermicularis*, *Galeoria hystrix*) and aragonitic structures (*Hydroides spongicola*).

## Discussion

### Taxonomic Implications

**Figure 5:**
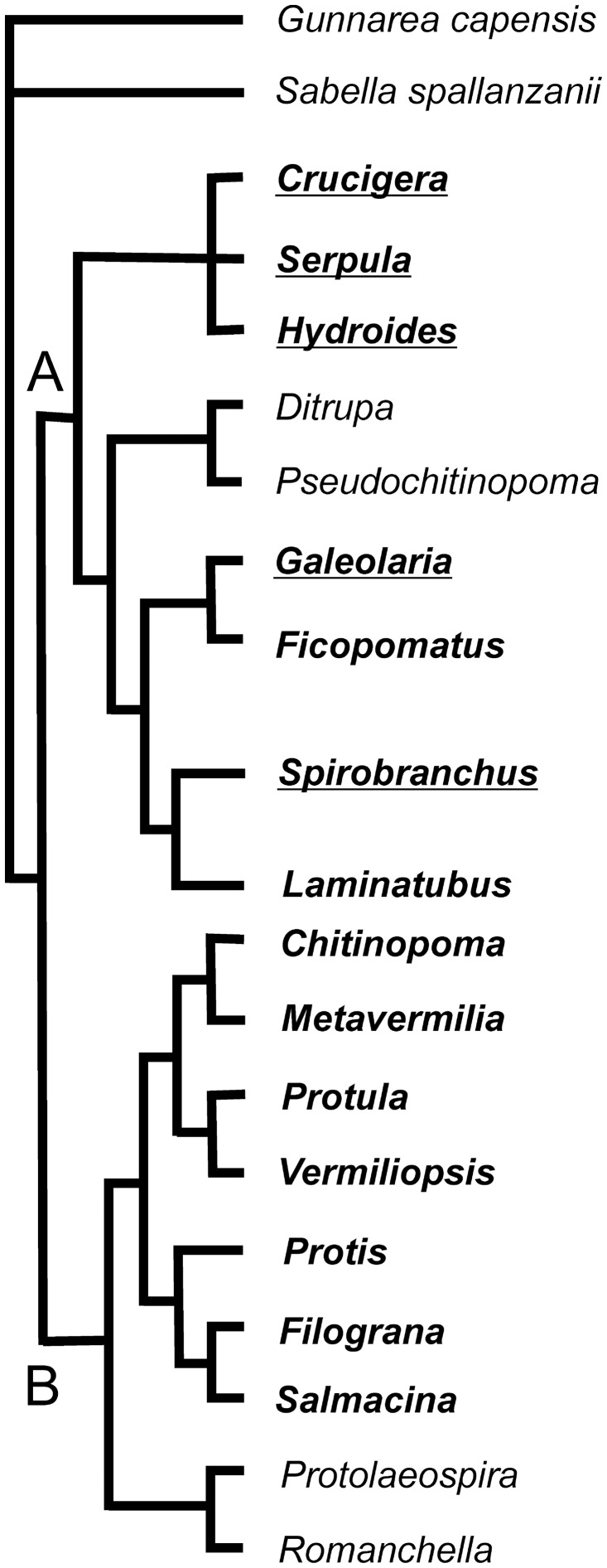
Evolution of organic sheets in Serpulidae. Phylogenetic relationships of serpulid genera are derived from Kupriyanova et(2006), Bayesian majority consensus cladogram of the combined molecular and morphological dataset. Bold letters-studied genera; Underlined- genera with organic sheets. A and B – two major clades of serpulids.

The organic sheets in the mineral tube structure occur only in certain taxa belonging to clade A (Figure 5), but not all species in clade A have them. Organic sheets are best developed in the genus *Spirobranchus*. They may also be characteristic at the generic level. Species differ in the abundance of organic sheets in their mineral layers of their tubes. Species also somewhat differ by the thickness of organic sheets (Table 1). However, the morphology of organic sheets does not allow distinguishing species in cross or longitudinal section of the tubes.

### Functions of Organic Sheets

The multiple tube layers with advanced complex microstructures of the species of clade A presumably have a tube strengthening function [6], [7]. One could speculate that the organic sheets possibly also represent an adaptation to strengthen the tube. The mechanical function of the organic sheets may be to make the tube wall more fracture-proof and less brittle by combining the strength of the biomineral layers with the elasticity of organic sheets. Another possible function of organic sheets may involve protection against the dissolution of the externally unprotected tubes made of CaCO_3_. In this case one would expect to find well developed organic sheets in deep sea serpulids where CaCO_3_-undersaturated waters could cause the dissolution of the mineral part of the tube. However, a deep sea species *Laminatubus alvini* (Clade A) does not have organic sheets, making the latter hypothesis dubious (Figure 5).

### Possible Formation and Relation to Mineral Structures

The morphological similarity and relatively similar dimensions of the organic sheets in the studied species presumably imply similar formation and function of these structures.

**Figure 6:**
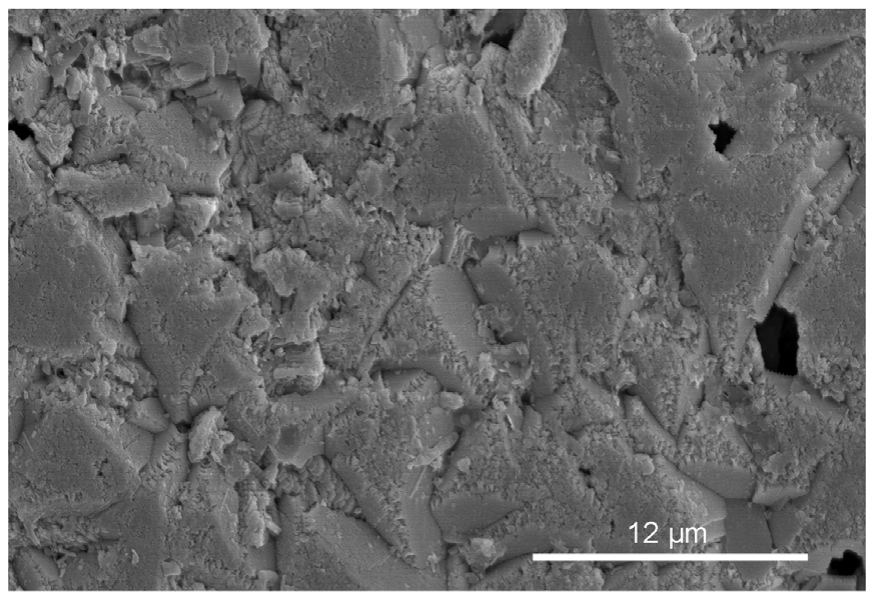
*Ditrupa arietina*, natural growth surface of prismatic crystals. Note the uneven nature of the growth surface.

The extremely flat growth surface of crystals of *Floriprotis sabiuraensis* (Figure 4) in contact with sheets implies that formation of sheets precedes the termination of crystallization in a growth increment. The termination of crystal growth without a preformed organic cover would result in uneven crystal growth surfaces, as with *Ditrupa arietina* (Figure 6, see [13]).

The irregularity of sheets in the tube structure indicates that the organic sheets are not always accompanied by the formation of a growth increment. In LF structure, organic sheets often do not separate growth increments with different crystal orientation. Most likely serpulids secrete episodically, but not regularly, calcareous mucus of a higher organic content, leading to the formation of organic sheets. It is also possible that the mucus used to form organic sheets contains different organic compounds than the mucus used to build mineral layers without organic sheets. The organic sheets in the mineral structures may have a similar composition to the inner organic tube lining. Tanur et al. [7] found that the inner organic lining of *Hydroides dianthus* was composed of collagen-containing fibres.

It is possible that the lower surface of the organic sheet terminates the crystal growth, while the upper surface may be used for crystal nucleation. Thus, it is also possible that the chemical properties of the lower and upper surface of the organic sheets could be different. In the case of prismatic structures, nucleated crystals grow epitaxially on the organic sheets. In contrast, in complex oriented structures the orientation of nucleated crystals is not controlled by epitaxy (the crystal orientation of the previous growth increment).

Organic sheets are probably not directly involved as a template in formation of complex oriented tube structures, as long segments (several increments with different crystal orientation) of LF structure could occur without any organic sheet in several species (i.e. *Serpula israelitica*, *S*. *vermicularis*, *Spirobranchus triqueter*).

### Evolution of Organic Sheets

Clade B contains only species with plesiomorphic tube microstructures such as IOP, SIOP and the other isotropic structures. The primitive serpulid tubes presumably had an inner organic lining and outer mineral layer with isotropic microstructure. The advanced, and probably apomorphic, oriented tube microstructures occur only in species of clade A. Organic sheets occur only in the clade A species with apomorphic advanced tube microstructures (Figure 5). It is possible that organic sheets represent an apomorphic character in the evolution of serpulid tubes. Thus, serpulids of clade A not only have more advanced mineral microstructures, but they also have more advanced insoluble organic matrix. It is difficult to reconstruct the evolution of organic sheets in clade A. They occur in most sub-clades of the clade but not in all of its genera (Figure 5). The question arises whether the organic sheets appeared once and then were lost in some species, or if they appeared multiple times in the evolution of clade A. The multiple appearances of organic sheets would seem more likely if the organic sheets do indeed represent a useful adaptation.

## Supporting Information

Table S1 **Serpulid species (studied) and their tube microstructures and occurrence of organic sheets.** Isotropic structures: HAC – homogeneous angular crystal structure, FH – fine grained homogeneous structure, IOP – irregularly oriented prismatic structure, IOPL – irregularly oriented platy structure, RHC – rounded homogeneous structure, SIOP – spherulitic irregularly oriented prismatic structure. Semi-oriented structures: SOIOP – semi-ordered irregularly oriented prismatic structure, SOSIOP – semi-ordered spherulitic oriented prismatic structure. Oriented prismatic structures: RRP- regularly ridged prismatic structure, SP – simple prismatic structure, SPHP – spherulitic prismatic structure. Oriented complex structures: LF – lamello-fibrillar structure, OF – ordered fibrillar structure, SLF – spherulitic lamello-fibrillar structure. Tube layers are ordered from outside (left) to lumen (right).
(DOC)
