# Supplementary material for: Occurrence, Formation and Function of Organic Sheets in the Mineral Tube Structures of Serpulidae (Polychaeta, Annelida)
Source: PLoS One. 2013 Oct 7;8(10):e75330. doi: 10.1371/journal.pone.0075330 (PMC3792063; doi:10.1371/journal.pone.0075330)
Supplement: Table S1 — Serpulid species (studied) and their tube microstructures and occurrence of organic sheets. Isotropic structures: HAC – homogeneous angular crystal structure, FH – fine grained homogeneous structure, IOP – irregularly oriented prismatic structure, IOPL – irregularly oriented platy structure, RHC – rounded homogeneous structure, SIOP – spherulitic irregularly oriented prismatic structure. Semi-oriented structures: SOIOP – semi-ordered irregularly oriented prismatic structure, SOSIOP – semi-ordered spherulitic oriented prismatic structure. Oriented prismatic structures: RRP- regularly ridged prismatic structure, SP – simple prismatic structure, SPHP – spherulitic prismatic structure. Oriented complex structures: LF – lamello-fibrillar structure, OF – ordered fibrillar structure, SLF – spherulitic lamello-fibrillar structure. Tube layers are ordered from outside (left) to lumen (right). (DOC) [file pone.0075330.s001.doc]

**Table S1.** Serpulid species (studied) and their tube microstructures and occurrence of organic sheets.

| **Species** | **Clade** | **Tube layers** | **Occurrence of organic sheets by tube layers/ thickness of sheets** | **Material studied (localities and depths, when known)** |
| --- | --- | --- | --- | --- |
| *Apomatus* *globifer* | B | IOP | Possible absence | V.Pol.0105.01 Kara Sea, 71°N, 64°E, 122 m |
| *Bathyvermilia langerhansi* | B | HAC/HAC | No | V.Pol.4041 Madeira, Porto Santo, St.4.180, 3499 m |
| *Chitinopoma* *serrula* | B | IOP | No | V.Pol.5034 Iceland, Snaefellsnes Peninsula, 30 m |
| *Chitinopomoides* *wilsoni* | B | SIOP | No | V.Pol.3166 Antarctica, USNM Acq.224443, 80 m |
| *Crucigera* *websteri* | A | SPHP/IOP/LF/SPHP | **IOP/LF/SPHP** (0.4-0.7 μm) | V.Pol.3589 Surinam, 60 m |
| *Crucigera* *zygophora* | A | SPHP/IOP/SIOP | Possible occurrence | V.Pol.3287 Canoe Bay, Alaska, USA, 8 m |
| *Ficopomatus* *enigmaticus* | A | IOP | No | V.Pol.3779 Lake of Tunis, 8 m |
| *Ficopomatus* *uschakovi* | A | IOP | No | V.Pol.3647 Thailand, Kong Prao, 1-2 m |
| *Filograna* *implexa* | B | IOP | No | V.Pol.3767 UK, Orkney Island, 22 m |
| *Filogranella elatensis* | B | IOP | No | V.Pol.3911 Japan, Sesoko Island, Okinawa, 10 m |
| *Filogranula gracilis* | B | IOP | No | V.Pol.4047 Cape Verde Islands, Boa Vista, 111 m |
| *Floriprotis* *sabiuraensis* | A | IOP/LF/SLF | **LF/SLF** (0.6-0.7 μm) | V.Pol.3929 Shimoshima Island, Amakusa, Japan, 10 m |
| *Galeolaria* *hystrix* | A | LF/SIOP | **LF/SIOP** (0.6-0.7 μm) | V.Pol.3576 New Zealand, Queen Charlotte Sound, 1-2 m |
| *Hyalopomatus* *marenzelleri* | A | IOP | No | V.Pol.4522 Canary Islands, Lanzarote,  1030-1070 m |
| *Hydroides dianthus* | A | SPHP/IOP/LF | LF (0.6-07 μm) | V.Pol.3661 USA, Anna Maria Island, FL, 2 m |
| *Hydroides* *spongicola* | A | SIOP | **SIOP** (0.5-0.7 μm) | V.Pol.3584 Netherlands Antilles, Curaçao, 7 m |
| *Janita* *fimbriata* | B | IOP | No | V.Pol.4072 Canary Islands, Lanzarote, 88 m |
| *Josephella* *marenzelleri* | B | IOP | No | V.Pol.3030 France, Marseille, depth unknown |
| *Laminatubus alvini* | A | HAC/IOP | No | V.Pol.3512 East Pacific Rise, 09°N, 104°W, 2509 m |
| *Marifugia* *cavatica* | A | IOP/IOP | No | V.Pol.3102 Hercegovina, Popovo Polje fresh waters, 1 m |
| *Metavermilia* *multicristata* | B | IOP | No | V.Pol.4300 Seychelles, N. of d’Arros Island, 55 m |
| *Neovermilia* *sphaeropomatus* | A | LF | Possible occurrence | V.Pol.3274 New Zealand, Cape Saunders, 10 m |
| *Paraprotis* *pulchra* | B | IOP | No | V.Pol.3379 Japan, Kushimoto, 70 m |
| *Placostegus* *tridentatus* | A | SP | Possible occurrence | V.Pol.1105 Norway, Bergensfjord |
| *Pomatostegus* *stellatus* | B | HAC | No | V.Pol.5170 Netherlands Antilles, Curaçao, 0.5 m |
| *Protis* *arctica* | B | IOP | No | V.Pol.3833 NE of Iceland, 1802 m |
| *Protula* *diomedeae* | B | SOIOP | No | V.Pol.4271 USA, Florida, 73 m |
| *Pseudovermilia* *madracicola* | B | SIOP | No | V.Pol.3751 Netherlands Antilles, Curaçao, Salinja Fuik, 27 m |
| *Pseudovermilia* *occidentalis* | B | IOP | No | V.Pol.4090 Cape Verde Islands, St. Luzia, 10 m |
| *Pyrgopolon* *ctenactis* | A | SOSIOP | Possible occurrence | V.Pol.4969 Netherlands Antilles, Bonaire, 15 m |
| *Rhodopsis* *pusilla* | B | IOP | No | V.Pol.3623 Reunion Island, 5 m |
| *Salmacina* *incrustans* | B | IOP | No | V.Pol.3814 Spain Costa Brava, 0.5 m |
| *Semivermilia* *crenata* | B | IOP | No | V.Pol.3045 France, Marseille, 1 m |
| *Serpula* *crenata* | A | SP/IOP | No | V.Pol.1739 Indonesia, 411 m |
| *Serpula israelitica* | A | LF | No | Cape Verde Islands, São Vicente, depth unknown |
| *Serpula* *vermicularis* | A | LF | LF (0.5-0.6 μm) | V.Pol.3780 Ireland, Ardbear Lough, 20 m |
| *Spiraserpula caribensis* | A | SPHP/SIOP/SPHP | No | Netherlands Antilles, Curaçao, 1 m |
| *Spirobranchus americanus* | A | IOP/LF | IOP/LF (0.6-0.7 μm) | ZMA V.Pol. 5009 Ensign, trawled 10 miles east of Bony, R’4’ (Knuckle Bony) off Cape Lookout Shoals, 10-20 m, U.S.A. |
| *Spirobranchus* *giganteus* | A | OF/SIOP | **OF/SIOP** (0.1-0.3 μm) | Netherlands Antilles, Curaçao, 6 m |
| *Spirobranchus kraussii* | A | SPHP/IOP/LF/SIOP | **IOP/LF/SIOP** (0.3-0.4 μm) | V.Pol.4748 Teluk Slawi, Indonesia, 0.5 m |
| *Spirobranchus triqueter* | A | LF | LF (0.4-0.5 μm) | Sweden, Tjärnö, 10 m |
| *Vermiliopsis* *infundibulum* | B | SIOP | No | V.Pol.4036 Canary Islands, Lanzarote, 80 m |
| *Vitreotubus digeronimoi* | A | SP | SP | V.Pol.4308, Seychelles, Platte Island, Sta. 795, 600 m |
